# Stop of proton-pump inhibitor treatment in patients with liver cirrhosis (STOPPIT): study protocol for a prospective, multicentre, controlled, randomized, double-blind trial

**DOI:** 10.1186/s13063-022-06232-w

**Published:** 2022-04-12

**Authors:** Malte H. Wehmeyer, Thomas Horvatits, Anika Buchholz, Linda Krause, Sarah Walter, Antonia Zapf, Ansgar W. Lohse, Johannes Kluwe, the STOPPIT-trial group

**Affiliations:** 1 I. Department of Medicine, University Medical Center Hamburg-Eppendorf, Hamburg, Germany; 2 Institute of Medical Biometry and Epidemiology, University Medical Center Hamburg-Eppendorf, Hamburg, Germany; 3 Coordinating Center for Clinical Trials Heidelberg, University Hospital Heidelberg, Heidelberg, Germany

**Keywords:** PPI, Pantoprazole, Esomeprazole, Side-effects, Complications, Liver cirrhosis, Infections, Hepatic encephalopathy, Mortality, Hospitalization

## Abstract

**Background:**

Proton-pump inhibitors (PPI) are liberally prescribed in patients with liver cirrhosis. Observational studies link PPI therapy in cirrhotic patients with an increased risk for infectious complications, hepatic encephalopathy and an increased risk for hospitalization and mortality. However, patients with liver cirrhosis are also considered to be at risk for peptic ulcer bleeding. The STOPPIT trial evaluates if discontinuation of a pre-existing PPI treatment delays a composite endpoint of re-hospitalization and/or death in patients (recently) hospitalized with liver cirrhosis compared to patients on continued PPI medication.

**Methods:**

The STOPPIT-trial is a prospective, multicentre, randomized, double-blinded, placebo-controlled, parallel-group trial. In total, 476 patients with complicated liver cirrhosis who already receive long-term PPI therapy without evidence-based indication are 1:1 randomized to receive either esomeprazole 20 mg (control group) or placebo (intervention group) for 360 days. Patients with an indication for PPI therapy (such as a recent diagnosis of peptic ulcers, severe reflux esophagitis, severe hemorrhagic gastritis, recent endoscopic therapy for oesophageal varices) are excluded. The primary composite endpoint is the time-to re-hospitalization and/or death. Secondary endpoints include rates of re-hospitalization, mortality, occurrence of infections, hepatic decompensation and acute-on-chronic liver failure. The safety endpoint is defined as manifestation of an evidence-based indication for PPI re-therapy. The impact of PPI continuation or discontinuation on the intestinal microbiota will be studied. The recruitment will take place at 18 study sites throughout Germany. Recruitment has started in April 2021.

**Discussion:**

The STOPPIT trial is the first clinical trial to study the effects of PPI withdrawal on relevant outcome variables in patients with complicated liver cirrhosis. If the hypothesis that PPI withdrawal improves clinical outcomes of cirrhosis patients is confirmed, this would argue for a strong restriction of the currently liberal prescription practice of PPIs in this population. If, on the other hand, the trial demonstrates an increased risk of gastrointestinal bleeding events in patients after PPI withdrawal, this could create a rationale for a more liberal, prophylactic PPI treatment in patients with liver cirrhosis.

**Trial registration:**

EU clinical trials register EudraCT 2019-005008-16 (registered December 27, 2019). ClinicalTrials.gov NCT04448028 (registered June 25, 2020). German Clinical Trials Register DRKS00021290 (registered March 10, 2021).

## Administrative information

Note: the numbers in curly brackets in this protocol refer to SPIRIT checklist item numbers. The order of the items has been modified to group similar items (see http://www.equator-network.org/reporting-guidelines/spirit-2013-statement-defining-standard-protocol-items-for-clinical-trials/).

**Table:** 

**Title {1}**	Stop of proton-pump inhibitor treatment in patients with liver cirrhosis – a double-blind, placebo controlled trial (STOPPIT)
**Trial registration {2a and 2b}**	EU clinical trials register EudraCT 2019-005008-16 (registered December 27, 2019) ClinicalTrials.gov NCT04448028 (registered June 25, 2020) German Clinical Trials Register DRKS00021290 (registered March 10, 2021)
**Protocol version {3}**	Protocol version 2.0, September 30, 2020
**Funding {4}**	The trial is funded by the German Federal Ministry of Education and Research (Bundesministerium für Bildung und Forschung, BMBF; grant no. FKZ 01KG2004).
**Author details {5a}**	Malte H. Wehmeyer, Thomas Horvatits, Johannes Kluwe, Ansgar W. Lohse: I. Department of Medicine, University Medical Center Hamburg-Eppendorf, Martinistraße 52, 20246 Hamburg, Germany. Anika Buchholz, Linda Krause, Antonia Zapf: Institute of Medical Biometry and Epidemiology, University Medical Center Hamburg-Eppendorf, Martinistraße 52, 20246 Hamburg, Germany. Sarah Walter: Coordinating Center for Clinical Studies, Heidelberg University Hospital, Berliner Straße 10, 69120 Heidelberg, Germany.
**Name and contact information for the trial sponsor {5b}**	University Medical Center Hamburg-Eppendorf Martinistraße 52, 20246 Hamburg, Germany Tel.: +4940-7410-0; Email: sponsor@uke.de
**Role of sponsor {5c}**	The trial protocol and patient information forms were reviewed for legal issues by sponsor representatives. Sponsor representatives provided advice with regard to trial management, risk-based monitoring and data protection. The sponsor had no influence on study design, as well as collection, management, analysis and interpretation of data, writing of the report or publication of the results.

## Introduction

### Background and rationale {6a}

Proton-pump inhibitors (PPI) are liberally and widely prescribed in patients with liver cirrhosis without a clear evidence-based indication [1–9]. Observational and retrospective studies suggest that PPI use in cirrhosis patients may be a risk factor for infections [10–14], especially spontaneous bacterial peritonitis (SBP) [8–10, 15, 16]. Increased SBP rates in cirrhotic patients may be explained through PPI-associated microbiotic shifts leading to small intestinal bacterial overgrowth and bacterial translocation [17–20]. PPI therapy was also suggested as a possible risk factor for pneumonia [10, 21, 22] and *Clostridium difficile* infections in cirrhotic patients [23]. However, other observational studies found no evidence for an association of PPI use and the risk of development of pneumonia [24], SBP [25–27] or infections in general [27]. Moreover, an association between episodes of (overt) hepatic encephalopathy and PPI use has been reported [6, 7, 9, 28, 29]. Infections and hepatic encephalopathy may often cause hospitalizations of cirrhotic patients. PPI use at discharge and PPI-mediated microbiotic shifts have also been associated to early re-hospitalization [20, 30, 31]. Also, PPI use has been reported to be an independent predictor for mortality in cirrhotic patients [5, 6, 10, 32, 33] and in a large North-American veteran cohort, as well as in patients with malignant diseases [34–36]. Again, other studies found no association between PPI use and mortality of cirrhotic patients [4, 27].

Thus, current evidence, even if not unambiguous, suggests an unfavourable risk profile of PPI in patients with liver cirrhosis. However, this patient population is considered to be at a high risk of gastrointestinal haemorrhage from peptic ulcers [37–40]. Importantly, peptic ulcer bleeding in patients with liver cirrhosis is associated with an increased mortality as compared to patients without cirrhosis [41–43], and mortality rates of cirrhotic patients with peptic ulcer bleeding are comparable to cirrhotic patients with bleeding from oesophageal varices [44]. Therefore, generous PPI use may also have a yet unproven preventive effect against upper gastrointestinal bleeding events. Short term use of PPIs for up to 10 days may reduce ulcer size after endoscopic variceal band ligation [45, 46], but there is no evidence for protective effects of PPI therapy against portal hypertension-related bleeding in cirrhotic patients who did not receive endoscopic ligation therapy [47].

### Objectives {7}

The primary objective of the trial is to determine the time to first unplanned re-hospitalization or death (composite endpoint) in patients with liver cirrhosis who discontinue long-term PPI therapy (intervention group) as compared to patients who continue PPI therapy (control group) over a period of 12 months (360 days). Secondary objectives include an assessment and comparison of the following endpoints in the two groups:

- Time to death and mortality (overall and liver-related),
- Time to and rate of unplanned re-hospitalization,
- Overall infection rates and infections rates differentiated by the site of infection,
- Rate of acute hepatic decompensation and acute-on-chronic liver failure (ACLF),
- Rate and source of upper and lower gastrointestinal bleeding events.

Furthermore, the changes in the intestinal microbiota in both groups and their impact on the primary endpoint will be assessed. The potential pharmacoeconomic impact of PPI discontinuation in patients with liver cirrhosis will be studied, too.

### Trial design {8}

**Fig. 1:**
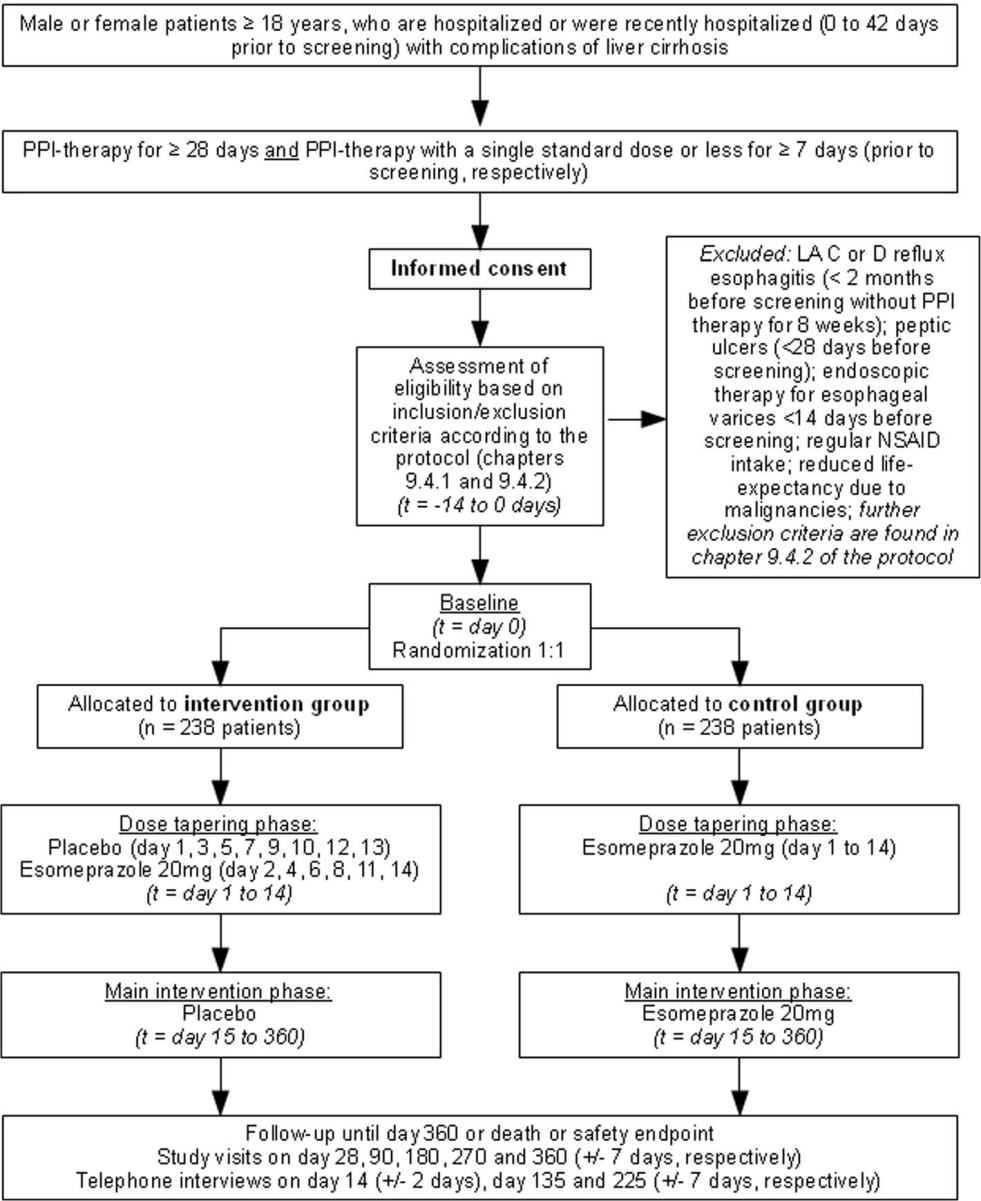
Flow chart of the STOPPIT trial. Please note, only key inclusion and exclusion criteria are depicted into this flow chart. Full in- and exclusion criteria are to be found in Table 1

The STOPPIT trial is an investigator-initiated, prospective, multicentre, randomized, placebo-controlled, double-blind, parallel-group trial. It is designed to demonstrate superiority of PPI discontinuation over PPI continuation in patients with liver cirrhosis. Figure 1 illustrates the trial design. In total, 476 patients will be randomized (1:1) to discontinue PPI therapy and receive placebo or to continue PPI therapy with esomeprazole 20 mg/day over a period of 360 days.

## Methods: participants, interventions and outcomes

### Study setting {9}

The clinical trial takes place in 18 university hospitals throughout Germany. The list of participating sites can be obtained on ClinicalTrials.gov (NCT04448028) and in the German Clinical Trials Registry (DRKS00021290). Each site has a high level of experience in the treatment of patients with liver cirrhosis and in conducting trials.

### Eligibility criteria {10}

Hospitalized, or recently hospitalized, patients with liver cirrhosis with a pre-existing long-term PPI therapy will be screened. Patients with an evidence-based indication for PPI therapy, for example, recent diagnosis of severe reflux esophagitis (LA grade C or D) or peptic ulcers are excluded (for details see Table 1).

**Table 1:** In- and exclusion criteria. Patients can be included in the trial, if they fulfil all the inclusion and none of the exclusion criteria

Inclusion criteria	Exclusion criteria
Male and female patients, at least 18 years old.	Diagnosis of reflux esophagitis LA grade C or D by EGD < 2 months prior to the screening visit without PPI therapy for at least 8 weeks prior to the screening visit.
Patients with liver cirrhosis. The diagnosis of liver cirrhosis may be based on (i) histology or (ii) a combination of clinical, laboratory and radiological criteria.	Peptic ulcers diagnosed by EGD < 28 days prior to the screening visit.
Hospitalization or recent hospitalization (0 to 42 days prior to the baseline visit) with complications of liver cirrhosis.	History of endoscopic therapy for oesophageal varices < 14 days prior to the screening visit.
Treatment with PPI for at least 28 days prior to screening.	Life-expectancy < 1 year (at the discretion of the investigator) due to extrahepatic malignancies, metastasized HCC, or other severe extrahepatic diseases (HCC without extrahepatic metastases or reduced life-expectancy < 1 year due to cirrhosis are not regarded as exclusion criteria).
Treatment with a PPI single standard dose/day or less for at least 7 days prior to screening.	Regular intake of NSAID on a daily basis (except for ASA 100 mg/day orally)
Females/males who agree to comply with the applicable contraceptive requirements.	One or more of the following measurements at the time of screening or documented up to 48 h before: ● Temperature > 38.5 °C ● Systolic blood pressure < 90 mmHg and heart rate > 100 bpm ● Catecholamine treatment > 0.1 μg/kg/min (terlipressin is allowed) ● Respiratory rate ≥ 22/min
Non-pregnant, non-lactating females.	Hypersensitivity or intolerance to esomeprazole, substituted benzimidazoles or other excipients of the IMP.
Ability to understand the patient information and to personally sign and date the informed consent to participate in the study, before completing any study-related procedures.	Ongoing therapy with nelfinavir.
The patient is co-operative and available for the entire study.	Participation in a clinical trial or use of an IMP within 30 days or five times the half-live of the IMP—whichever is longer—prior to receiving the first dose within this study.
Provided written informed consent.	Positive urine pregnancy test at screening or positive serum pregnancy test before the first treatment or is breast feeding.
	Patient is not willing to use adequate contraceptive precautions during the study and up for 5 days after the last scheduled dose of IMP.

*EGD* oesophago-gastro-duodenoscopy, *PPI* proton-pump inhibitors, *HCC* hepatocellular carcinoma, *NSAID* non-steroidal anti-inflammatory drugs, *ASA* acetylsalicylic acid, *IMP* investigational medicinal product

### Who will take informed consent {26a}

The principal investigator or a trained and designated physician has to obtain written informed consent from the patient prior to any study-related procedures. Patients will first have ample time to read the patient information. An investigator will provide the patients with information on the study. After the patient had ample time for consideration and formulation of questions (which could also mean that the patients first discuss the decision with friends or family members), and if all questions are answered the patient will be asked to personally sign the informed consent form. A copy or a second original of the signed informed consent form must be given to the patients for their records.

### Additional consent provisions for collection and use of participant data and biological specimens {26b}

Study participants have the option to consent on the collection of additional biobanking samples. Importantly, if the study participant does not provide informed consent for biobanking, the patient can still participate in the main study.

## Interventions

### Explanation for the choice of comparators {6b}

Patients in the intervention group discontinue a pre-existing long-term PPI therapy and replace it with placebo, while the pre-existing PPI treatment in the control group is replaced with esomeprazole 20 mg/day. Esomeprazole 20 mg was chosen as the comparator, because it is the only PPI which is assumed as “safe” for patients with advanced cirrhosis according to the available data on pharmacokinetics [48]. Moreover, 20 mg esomeprazole is equivalent to a PPI standard dose [49] and therefore adequately reflects the “common use” of PPI medication in clinical routine.

### Intervention description {11a}

All patients discontinue their previous PPI therapy during the baseline visit to start study medication on day 1 of the trial: Patients randomized to the intervention group receive placebo (P-Tablette weiß 7 mm Lichtenstein, Winthrop Arzneimittel GmbH, Frankfurt am Main, Germany) and patients randomized to the control group receive esomeprazole 20 mg/day (Nexium mups, Grünenthal GmbH, Aachen, Germany) over a period of 360 days. To prevent patients from gastric acid rebound symptoms caused by sudden PPI withdrawal, study participants will undergo a dose tapering phase over a period of 14 days in which they take the study medication from drug dispensers (“trial starter drug dispenser”; intervention group: placebo on days 1, 3, 5, 7, 9, 10, 12, and 13 and esomeprazole on days 2, 4, 6, 8, 11, and 14; control group: esomeprazole 20 mg days 1 to 14). After that, the patient will take the trial medication from “study medication packages” each containing 100 capsules placebo or esomeprazole 20 mg, respectively. Patients are provided with the “trial starter drug dispenser” and one “study medication package” during the baseline visit (visit 1). Furthermore, they receive a “study medication package” at visit 3 (day 90), 4 (day 180) and 5 (day 270).

### Criteria for discontinuation or modifying allocated interventions {11b}

Patients who reach the safety endpoint (“evidence-based indication for re-therapy with PPI”; Table 2) will discontinue study medication. However, in case of clinically suspected peptic ulcer disease or signs of upper gastrointestinal haemorrhage rescue PPI treatment is permitted for up to 72 h or until an oesophago-gastro-duodenoscopy (EGD) is performed. If the EGD does not reveal a safety endpoint, rescue PPI therapy will be stopped and the patient continues study medication.

**Table 2:** Primary, secondary and safety endpoints of the trial

Primary composite endpoint	Secondary endpoint
1.) Unplanned re-hospitalization	1.) Death
2.) Death	2.) Unplanned re-hospitalization
	3.) Any infection and differentiated by site of infection (SBP, pneumonia, urinary tract infection, blood stream infection, Clostridium difficile-associated enterocolitis, Norovirus infection, SARS-CoV-2 infection, others)
	4.) Acute hepatic decompensation and ACLF
	5.) Upper or lower gastrointestinal bleeding event
	**Safety endpoint:** Evidence based re-therapy with PPI due to the occurrence of any the following conditions:
	1.) Peptic ulcer diagnosed by EGD
	2.) Reflux esophagitis LA grade C or D diagnosed by EGD
	3.) Severe hemorrhagic gastritis diagnosed by EGD and histology
	4.) Mallory-Weiss syndrome diagnosed by EGD

*SBP* spontaneous bacterial peritonitis, *SARS-CoV-2* severe acute respiratory syndrome coronavirus 2, *ACLF* acute-on-chronic liver failure, *PPI* proton-pump inhibitors, *EGD* oesophago-gastro-duodenoscopy

### Strategies to improve adherence to interventions {11c}

Drug accountability measures and patient diaries are implemented to improve protocol adherence. To avoid protocol violations due to dyspeptic and/or reflux symptoms after sudden discontinuation of PPI in the intervention group, patients undergo PPI dose tapering. Dyspepsia or symptomatic gastroesophageal reflux disease may be treated with sucralfat. Study participants are reminded to take study medication from the “study medication package” from day 15 on during a telephone interview on day 14.

### Relevant concomitant care permitted or prohibited during the trial {11d}

Open-label PPIs, h2-receptor antagonists and nelfinavir are prohibited during this trial (exception: rescue PPI therapy for up to 72 h in patients with suspected upper gastrointestinal bleeding events or peptic ulcers). Non-steroidal anti-inflammatory drugs (other than acetylsalicylic acid up to 100 mg/day) for more than 7 days during a period of 28 days are not permitted. Treatment with sucralfat for dyspepsia or symptomatic reflux disease is permitted.

### Provisions for post-trial care {30}

After completion of the trial, participants will be treated according to the current guidelines in the respective trial centres. All study participants are provided with an insurance to cover trial-related medical issues.

### Outcomes {12}

The primary composite endpoint is the time to unplanned re-hospitalizations or death during the trial period. However, expected re-hospitalization for mere paracentesis (without other complication of cirrhosis) during a period of 30 days after discharge is not regarded as an “unplanned re-hospitalization”. If the local investigator and the clinical trial management come to a conflicting assessment whether a re-hospitalization was “planned” or “unplanned”, a blinded endpoint committee (consisting of three independent hepatologists) will resolve this disagreement.

Secondary endpoints are described in Table 2. Importantly, patients who reach the primary endpoint “re-hospitalization” will continue all study procedures and will further receive the study medication. Therefore, secondary endpoints will be studied after a study participant has reached the primary endpoint. Patients who reach the safety endpoint (evidence-based re-therapy with PPI; see Table 2) are censored at the respective time-point.

### Participant timeline {13}

Recruitment is planned to be completed within three years. With a trial duration 360 days per patient, time of first-patient-in to last-patient-out is four years. Figure 1 provides an overview of the trial design. After completion of the informed consent process, the screening visit will take place. Randomization will take place 0 to 14 days after screening during the baseline visit (visit 1, day 0). The study is split into two parts: “dose tapering phase” (days 1–14; tapering of the PPI dose in a blinded fashion, see above) and “main study phase” (days 15–360). Study visits and telephone interviews take place at the time-points indicated in Table 3. Study procedures during the respective visits and telephone interviews are summarized in Table 4.

**Table 3:** Visits and time points

Screening visit	Up to 14 days before baseline
**Visit 1 (baseline)**	Day 0
**Visit 2**	Day 28 (± 2 days)
**Visit 3, 4, 5, 6**	Day 90, 180, 270, 360 (± 7 days, respectively)
**Telephone interview 1**	Day 14 (± 2 days)
**Telephone interview 2, 3**	Day 135, 225 (± 7 days, respectively)

**Table 4:** Study procedures

Procedure	Screening	Baseline visit	Visits 2–6	Telephone interviews
Informed consent	X			
In-/exclusion criteria	X			
Randomization		X		
Medical history/demographics	X	X		
Adverse events/concomitant medication/endpoint assessment		X	X	X
Physical examination/height and weight		X	X	
Vital sign assessment	X	X	X	
Laboratory (haematology, biochemistry)		X	X	
Pregnancy test	X			
Stool samples & biobanking		X	X (visit 3)	
Ultrasound		X	X (visit 4, 6)	
Distribution and/or return of study medication and patients diaries		X	X	

### Sample size {14}

The sample size is based on the composite primary endpoint time to death from any cause or time to unplanned re-hospitalization from any cause. Based on previous studies on mortality in cirrhosis patients who receive PPI [5, 6, 10, 32, 33] and an assumed 50% risk for re-hospitalization 3 months after discharge in decompensated cirrhotic patients [20, 30, 50], it is expected that the control group will have a probability of 65% to reach the primary endpoint after 6 months. The risk to reach the primary endpoint in the PPI withdrawal group is assumed to be 50% after 6 months, due to a reduction of mortality [4–6, 8, 10, 11] and a decreased risk for infectious complications [8–10, 12–16], which results in a lower risk for re-hospitalization [20]. This corresponds to a HR of 0.66 of the intervention group compared to the control group. The sample size calculation is performed on the assumed event rates of 50% in the intervention group (PPI withdrawal) and 65% for the control group after 6 months with a two-sided significance level α of 5% and a power of 80%, which requires a total number of 184 events to be observed. With a follow-up period of exactly 360 days per patient and an estimated 20% random loss to follow-up or non-compliance it can safely be assumed that a sufficient number of events will have been observed by the end of the trial if a total of 475 patients are available for analysis (based on the log-rank test; PASS 16.0.3). Therefore, 476 patients will be randomized.

### Recruitment {15}

Patients will be recruited at the trial centres during or after hospitalization. Global and local recruitment rates will be monitored by the clinical project management and the data safety and monitoring board (DSMB). Trial sites are obliged to recruit a number of patients according to their respective pre-study prognosis. In case trial sites do not fulfil these obligations in a specified time, countermeasures can be taken (e.g., direct communication with the investigator, addendum to the protocol).

## Assignment of interventions: allocation

### Sequence generation {16a}

Randomization will be carried out locally. A block randomization procedure with variable block length will be applied stratified for the study centres and severity of liver cirrhosis (MELD score ≤13 or > 13). Blinding of therapy for the patients and the investigators will be ensured by identical encapsulation of placebo and esomeprazole 20 mg that makes both indistinguishable, and by the use of identical medication packages. Unique randomization codes labelled on the medication packages ensure the specific allocation of the study medication to the study participant.

### Concealment mechanism {16b}

Each centre has two randomization lists (MELD ≤13 and > 13). The lists include only the randomization numbers and do not reveal the study medication.

### Implementation {16c}

Only the Pharmacy of the University Medical Center Hamburg-Eppendorf performing the randomization procedure, and neither the investigators nor the study participants, will be aware of the allocation sequence or the block size used. Randomization numbers are allocated to patients sequentially by local study staff.

## Assignment of interventions: blinding

### Who will be blinded {17a}

Investigators, study participants, (clinical) trial management, data management, pharmacovigilance officers and data analysts are blinded. Only the central pharmacy and the data safety monitoring board (DSMB) members are unblinded.

### Procedure for unblinding if needed {17b}

Access to emergency envelopes is regulated at all trial sites. Details on the unblinding procedures are found in the investigator site files.

## Data collection and management

### Plans for assessment and collection of outcomes {18a}

Clinical outcome, baseline and other trial data will be collected during the visits and telephone interviews (Table 4) at the time points detailed in Table 3. Site staff will transfer trial data from the source documents into the electronic case report form (eCRF) and check eCRF entries for completeness. Corrections to source data or eCRF data will be dated and signed. Reasons for changes must be provided. Source data verification will take place during on-site monitoring. To initiate discrepancy resolution the monitor will send queries to the site staff.

### Plans to promote participant retention and complete follow-up {18b}

If possible, complete follow-up data should be obtained from all patients. Investigators motivate trial participants to attend all study visits. Patients who discontinue the investigational product will attend all subsequent study visits, in which endpoint data will be collected. The primary efficacy analysis will follow intention-to-treat principles.

### Data management {19}

Data management will check predefined eCRF entries as defined in the data validation plan. Quality control and data validation procedures such as programmed automatic edit and consistency checks ensure data validity and accuracy immediately at the point of entry into the clinical database. The database application is an access restricted, demands electronic signatures, maintains an electronic audit trail and provides appropriate backup functionalities.

### Confidentiality {27}

Data obtained during the trial will be treated pursuant to the Federal Data Protection Law. Subjects will be identified solely by their individual randomization number. Trial data stored on a computer will be stored in accordance with local data protection law and will be handled in the strictest confidence. The appropriate regulations of local data legislation will be fulfilled in its entirety.

### Plans for collection, laboratory evaluation and storage of biological specimens for genetic or molecular analysis in this trial/future use {33}

Blood and urine samples will be collected for biobanking and further analysis in the future. Patients may choose not to provide these optional biobanking samples.

## Statistical methods

### Statistical methods for primary and secondary outcomes {20a}

The primary efficacy endpoint analysis will be performed according to the intention-to-treat (ITT) principle based on the full analysis set (FAS). The effects of continuation or discontinuation of PPI therapy with respect to the primary endpoint will be estimated and tested by Cox regression. The regression model will include treatment arm and categorized the severity of cirrhosis (MELD ≤ 13 vs. > 13) as fixed effects and the study centre as a random effect. As an estimated effect size, the hazard ratio (HR) between the two treatment arms will be given with the corresponding 95% confidence interval. The one-sided test of the superiority of discontinuation of PPI over the continuation of PPI at significance level 2.5% will be based on the corresponding asymptotic two-sided 95% confidence interval from the Cox regression model. The null hypothesis is rejected if the upper limit of the confidence interval is below 1.0. The analysis of the treatment effect with respect to the individual components of the composite endpoint and to secondary endpoints will be performed analogously with the (cause-specific) Cox model. Time to event outcomes will be visualized by Kaplan-Meier curves stratified by treatment group. Adverse event data will be summarized by the treatment group.

### Interim analyses {21b}

After half of the required events, an interim analysis is planned with the opportunity to stop for futility if the p-value of the treatment group is > 0.5. Since this interim analysis does not inflate the type-one error α correction for multiplicity is not necessary.

### Methods for additional analysis (e.g. subgroup analyses) {20b}

The composite primary endpoint will additionally be analysed descriptively by applying a multi-state model with states of unplanned re-hospitalization and death. Both analyses of the composite primary endpoint will be repeated in the per-protocol (PP) set. Analyses of the primary and secondary endpoint in the following subgroups are planned: According to the Child-Pugh stage; according to the aetiology of cirrhosis; untreated or treated aetiology of cirrhosis (e.g. treated vs. untreated viral hepatitis); patients with a history of TIPS implantation. Further subgroups may be defined in ad-hoc analyses.

### Methods in analysis to handle protocol non-adherence and any statistical methods to handle missing data {20c}

In the primary analysis, dropout will be dealt with as independent right censoring. In case of substantial dropout, this assumption will be investigated in sensitivity analyses, e.g. shared random effects models. Missing data in baseline variables will be handled by multiple imputation.

### Plans to give access to the full protocol, participant level-data and statistical code {31c}

Access to the full protocol and statistical code is possible on reasonable request after approval by the executive committee. Anonymized participant-level data are available after the publication of the primary results on reasonable request after approval by the executive committee.

## Oversight and monitoring

### Composition of the coordinating centre and trial steering committee {5d}

Professor Dr. Ansgar W. Lohse is the coordinating investigator. PD Dr. Johannes Kluwe is the deputy coordinating investigator. Dr. Malte H. Wehmeyer and Dr. Thomas Horvatits are responsible for clinical trial management and medical monitoring. Professor Dr. Antonia Zapf is the responsible trial statistician. All five are members of the trial executive committee (Chair: Prof. Dr. Lohse).

### Composition of the data monitoring committee, its role and reporting structure {21a}

A Data Safety Monitoring Board (DSMB) is constituted to protect the safety of study participants. It consists of three members (two hepatologists and one statistician) and will be scheduled at least every 6 months. It assesses the progress, safety data, and critical efficacy endpoints of the trial, as well as external factors (e.g. scientific results) which might affect participant safety or ethical status. The DSMB will receive blinded data and unblinded data (to be reviewed in a closed session). Based on the observed benefits or adverse effects, the DSMB will make recommendations to the sponsor concerning continuation, termination or modification of the trial. The sponsor has established a charter document explaining the working procedures for the DSMB.

### Adverse events reporting and harms {22}

An adverse event (AE) is defined as any untoward medical occurrence in a study participant, which does not necessarily have a causal relationship with the study treatment. AEs will be ascertained by the investigators during visits and telephone interviews using non-leading questions, noted as spontaneously reported by the patients to the medical staff or observed during any measurements during the trial. All AEs will be reviewed, confirmed, and classified by an investigator. All subjects who present AEs will be monitored by the responsible investigator to determine their outcome (also in patients who were withdrawn from the trial). AEs are classified as “serious” or “non-serious”, all AEs are graded (mild, moderate, severe) and the relationship between the study drug and outcome is documented. The action taken with the study drug and countermeasures have to be recorded for each AE.

Serious adverse events (SAE) are defined as AEs resulting in death, are life-threatening, require hospitalization or prolongation of existing hospitalization, result in persistent or significant disability/incapacity, are a congenital anomaly/birth defect or are otherwise medically relevant. All SAEs must be reported by the investigator to the pharmacovigilance department within 24 h after the SAE becomes known. All SAEs will be subject to a second assessment by a designated person independent from the reporting investigator. SAEs that are potentially attributed to the study drug are classified as serious adverse reactions (SAR). For each SAR, “expectedness” has to be assessed. All suspected unexpected SAR (SUSAR) are subject to expedited reporting to the responsible ethics committees, the competent authorities and to all participating investigators. Pregnancies of participants during the trial have to be reported the same way as SAEs (but are not regarded as a SAE).

### Frequency and plans for auditing trial conduct {23}

The project management group (including clinical trial management, non-clinical trial management, pharmacovigilance officers, database officers and clinical research associates) meets monthly to assess recruitment, protocol deviations and SAEs. Furthermore, the clinical trial management team meets on a weekly basis to discuss patient recruitment and SAEs. Furthermore, risk-based quality management meetings (RBQM) are scheduled twice a year to possibly adjust monitoring strategies. All sites are regularly monitored by a clinical research associate. Details with regard to the meetings of the Data Safety Monitoring Board (DMSB) are provided above {item 21a}.

Standard phases of the study may be subject to audits by an independent party authorised by the Sponsor. Results of these audits as well as any objections will be reported directly to the Sponsor. Audits will be planned on demand.

### Plans for communicating important protocol amendments to relevant parties (e.g. trial participants, ethical committees) {25}

Modifications of the protocol must be authorised by the sponsor and the coordinating investigator. Deviations and changes to the study protocol will be classified as:

- Note-to-File: Clarifications which are not considered changes of the protocol.
- Study protocol amendment: Substantial changes of the protocol, which need approval from the ethics committees and competent authority. These changes may also induce revision of the informed consent form. Patients undergoing trial assessment procedures at the time of implementation of the change have to be given the amended version and have to be asked for consent to continue on the trial.

### Dissemination plans {31a}

Study results will be presented at international meetings and submitted to a high impact, peer-reviewed scientific journal (open access) 3 months after the end of data analysis. The reporting will follow the recommendations of the CONSORT group for the reporting of randomized clinical trials. Authorship eligibility guidelines for publications are defined in the full protocol. Furthermore, final results will be provided to clinicaltrials.gov and the EudraCT database.

## Discussion

Prophylactic long-term PPI treatment without evidence-based indication is frequently regarded as “common use” in daily clinical routine in patients with liver cirrhosis [1–9]. Increasing evidence from observational studies suggests possible harmful effects of chronic PPI treatment in cirrhotic patients. However, the negative impact of chronic PPI treatment on infection rates [8–16, 21–23], rates of hepatic decompensation [6, 7, 9, 28, 29], hospitalization rates [20, 30, 31] and mortality of cirrhotic patients [5, 6, 10, 32, 33] remains controversial due to conflicting data [4, 24–27]. Therefore, it remains unclear if PPI use is causative for patient mortality or merely associated with comorbidities which themselves lead to a higher mortality in patients [51, 52]. On the other hand, patients with liver cirrhosis are at an increased risk of variceal and nonvariceal gastrointestinal bleedings [37–44] and PPI could have preventive effects.

The primary aim of this study is to prove superiority of PPI discontinuation over continuation in (recently) hospitalized liver cirrhosis patients without evidence-based indication for PPI use with regard to the composite primary endpoint “death and/or unplanned re-hospitalization”. As the above-mentioned complications regularly lead to a hospitalization of cirrhotic patients and a hospitalization itself is considered a risk factor for mortality in patients with liver cirrhosis [50], re-hospitalization is an adequate surrogate for patient-relevant complications of liver cirrhosis. By aggregation of unplanned re-hospitalization and mortality to a composite endpoint, we avoid underestimation of clinically significant events resulting in pre-clinical mortality. However, this implies a risk that mortality will be underrepresented in the primary results, as most fatal outcomes are expected to occur after re-hospitalization. Thus, incidence of secondary endpoints including mortality will be studied after re-hospitalization. We will not regard expected re-hospitalization due to ascites (without SBP or other complications) during a period of 30 days after discharge as a primary end-point criterion, as patients with refractory ascites are often expected to be re-hospitalized for paracentesis.

Importantly, sudden PPI withdrawal in the intervention group may lead to clinically relevant overproduction of gastric acid with a recurrence of dyspeptic symptoms [53–55], which could lead to an increased risk for protocol violations (like the intake of PPIs or other acid-suppressive drugs). Therefore, PPI discontinuation will be achieved after dose tapering as previous data suggest a clinical benefit of dose tapering instead of abrupt withdrawal of PPI therapy [55].

In- and exclusion criteria are designed to ensure that the study population represents all patients with complicated liver cirrhosis in whom PPI discontinuation is feasible and may have positive effects on patients’ outcome: Especially hospitalized patients with complications of liver cirrhosis are at risk for re-hospitalization [20, 30, 56] and hospitalization itself is associated with increased mortality in this population [50]. Furthermore, the incidence of an infection (which is regarded a complication of cirrhosis in our protocol) is often a deteriorating event in the course of cirrhotic disease leading to ACLF [57]. As the study aims to evaluate the discontinuation of widely practiced “prophylactic” long-term PPI prescription without strong indication, a PPI therapy has to be documented for ≥ 28 days prior to screening. However, only patients taking a single PPI standard dose per day or less for at least 7 days are included in the trial, as patients receiving higher doses of PPI might be at an increased risk to suffer from gastric acid rebound symptoms [53–55] despite our PPI dose tapering protocol.

The exclusion criteria represent evidence-based indications for PPI therapy also in cirrhosis patients according to the German guidelines [58–60]. In these patients, discontinuation of PPI therapy might be harmful. Patients who underwent endoscopic therapy for oesophageal varices < 14 days prior to screening are also excluded, as PPI therapy may reduce the size of banding-ulcers after variceal band ligation [45, 46]. Patients with a life-expectancy below 1 year due to extrahepatic diseases or malignancies are excluded to avoid confounding of the primary efficacy endpoint, as these patients have a high risk to reach the primary endpoint independently from cirrhosis or the trial intervention. Also, patients who are regarded clinically unstable are excluded from the trial.

In conclusion, the STOPPIT trial represents the first prospective, randomized, placebo-controlled trial which aims to confirm the expected benefit of a discontinuation of long-term PPI therapy in cirrhotic patients. If the primary hypothesis (superiority of PPI discontinuation over continuation of PPI therapy) is confirmed, the trial will have significant impact on the recommended drug management of patients with liver cirrhosis. Reduced PPI prescription rates could lead to lower rates of cirrhosis-related complications and may have a positive impact on (re-)hospitalization rates and mortality. Furthermore, PPI discontinuation would possibly lower the “pill burden”, lower the risk of drug-drug interactions in cirrhotic patients, and may therefore have a positive socioeconomic impact.

## Trial status

The information presented in this article are based on the STOPPIT-Study protocol version 2.0 (September 30, 2020). Recruitment has started in April 2021 (*first patient in*) and is expected to be completed by March 2024 (*last patient in*).

## Abbreviations

ACLF: Acute-on-chronic liver failure
AE: Adverse event
DSMB: Data safety monitoring board
eCRF: Electronic case report form
EGD: Oesophago-gastro-duodenoscopy
FAS: Full analysis set
HR: Hazard ratio
MELD: Model for end-stage liver disease
PP: Per-protocol
PPI: Proton-pump inhibitors
SAE: Serious adverse event
SAR: Serious adverse reaction
SBP: Spontaneous bacterial peritonitis
SUSAR: Suspected unexpected serious adverse reaction
TIPS: Tranjugular intrahepatic portosystemic shunt

## References

[1] D'Amico G, Garcia-Tsao G, Pagliaro L (2006). Natural history and prognostic indicators of survival in cirrhosis: a systematic review of 118 studies. J Hepatol.

[2] Johnson DA, Oldfield EC (2013). Reported side effects and complications of long-term proton pump inhibitor use: dissecting the evidence. Clin Gastroenterol Hepatol.

[3] Horvatits T, Drolz A, Wehmeyer M, Steib C, Trebicka J, Lohse AW, Kluwe J (2019). Proton pump inhibitors in patients with liver cirrhosis – a survey among hepatologists in Germany. Z Gastroenterol.

[4] Cole HL, Pennycook S, Hayes PC (2016). The impact of proton pump inhibitor therapy on patients with liver disease. Aliment Pharmacol Ther.

[5] Dultz G, Piiper A, Zeuzem S, Kronenberger B, Waidmann O (2015). Proton pump inhibitor treatment is associated with the severity of liver disease and increased mortality in patients with cirrhosis. Aliment Pharmacol Ther.

[6] Nardelli S, Gioia S, Ridola L, Farcomeni A, Merli M, Riggio O (2019). Proton pump inhibitors are associated with minimal and overt hepatic encephalopathy and increased mortality in patients with cirrhosis. Hepatology.

[7] Sturm L, Bettinger D, Giesler M, Boettler T, Schmidt A, Buettner N, Thimme R, Schultheiss M (2018). Treatment with proton pump inhibitors increases the risk for development of hepatic encephalopathy after implantation of transjugular intrahepatic portosystemic shunt (TIPS). United European Gastroenterol J.

[8] Bajaj JS, Zadvornova Y, Heumann DM (2009). Association of proton pump inhibitor therapy with spontaneous bacterial peritonitis in cirrhotic patients with ascites. Am J Gastroenterol.

[9] Dam G, Vilstrup H, Watson H, Jepsen P (2016). Proton pump inhibitors as a risk factor for hepatic encephalopathy and spontaneous bacterial peritonitis in patients with cirrhosis with ascites. Hepatology.

[10] Dam G, Vilstrup H, Andersen PK, Bossen L, Watson H, Jepsen P (2019). Effect of proton pump inhibitors on the risk and prognosis of infections in patients with cirrhosis and ascites. Liver Int.

[11] Merli M, Lucidi C, Di Gregorio V (2015). The chronic use of beta-blockers and proton pump inhibitors may affect the rate of bacterial infections in cirrhosis. Liver Int.

[12] Bajaj JS, Ratliff SM, Heumann DM (2012). Proton pump inhibitors are associated with a high rate of serious infections in veterans with decompensated liver cirrhosis. Aliment Pharmacol Ther.

[13] O’Leary JG, Reddy KR, Wong F (2015). Long-term use of antibiotics and proton pump inhibitors predict development of infections in patients with cirrhosis. Clin Gastroenterol Hepatol.

[14] Sargenti K, Kalaitzakis E (2015). Subsequent bacterial infections in patients with cirrhosis and the role of proton-pump inhibitors. Clin Gastroenterol Hepatol.

[15] Min YW, Lim KS, Min BH, Gwak GY, Paik YH, Choi MS, Lee JH, Kim JJ, Koh KC, Paik SW, Yoo BC, Rhee PL (2014). Proton pump inhibitor use significantly increases the risk of spontaneous bacterial peritonitis in 1965 patients with cirrhosis and ascites: a propensity score matched cohort analysis. Aliment Pharmacol Ther.

[16] Deshpande A, Pasupuleti V, Thota P, Pant C, Mapara S, Hassan S, Rolston DDK, Sferra TJ, Hernandez AV (2013). Acid-suppressive therapy is associated with spontaneous bacterial peritonitis in cirrhotic patients: a meta-analysis. J Gastroenterol Hepatol.

[17] Lo WK, Chan WW (2013). Proton pump inhibitor use and the risk of small intestinal bacterial overgrowth: a metaanalysis. Clin Gastroenterol Hepatol.

[18] Bajaj JS, Cox IJ, Bertrapally NS (2014). Systems biology analysis of omeprazole therapy in cirrhosis demonstrates significant shifts in gut microbiota composition and function. Am J Physiol Gastrointest Liver Physiol.

[19] Yamamoto K, Ishigami M, Honda T, Takeyama T, Ito T, Ishizu Y, Kuzuya T, Hayashi K, Goto H, Hirooka Y (2019). Influence of proton pump inhibitors on microbiota in chronic liver disease patients. Hepatol Int.

[20] Bajaj JS, Acharya C, Fagan A, White MB, Gavis E, Heuman DM, Hylemon PB, Fuchs M, Puri P, Schubert ML, Sanyal AJ, Sterling RK, Stravitz TR, Siddiqui MS, Luketic V, Lee H, Sikaroodi M, Gillevet PM (2018). Proton pump inhibitor initiation and withdrawal affects gut microbiota and readmission risk in cirrhosis. Am J Gastroenterol.

[21] Laheij RJ, Sturkenboom MC, Hassing RJ, Dieleman J, Stricker BH, Jansen JB (2004). Risk of community-acquired pneumonia and use of gastric acidsuppressive drugs. JAMA.

[22] Lambert AA, Lam JO, Paik JJ, Ugarte-Gil C, Drummond MB, Crowell TA (2015). Risk of community-acquired pneumonia with outpatient proton-pump inhibitor therapy: a systematic review and meta-analysis. PLoS One.

[23] Yan D, Huang YD, Chen YB, Lv T, Gu SL, Li YT, Huang JR, Li LJ (2019). Risk factors for Clostridium difficile infection in cirrhotic patients. Hepatobiliary Pancreat Dis Int.

[24] Dublin S, Walker RL, Jackson ML, Nelson JC, Weiss NS, Jackson LA (2010). Use of proton pump inhibitors and H2 blockers and risk of pneumonia in older adults: a population-based case-control study. Pharmacoepidemiol Drug Saf.

[25] Terg R, Casciato P, Garbe C, Cartier M, Stieben T, Mendizabal M, Niveyro C, Benavides J, Marino M, Colombato L, Berbara D, Silva M, Salgado P, Barreyro F, Fassio E, Gadano A (2015). Proton pump inhibitor therapy does not increase the incidence of spontaneous bacterial peritonitis in cirrhosis: a multicenter prospective study. J Hepatol.

[26] Kim JH, Lim KS, Min YW, Lee H, Min BH, Rhee PL, Kim JJ, Koh KC, Paik SW (2017). Proton pump inhibitors do not increase the risk for recurrent spontaneous bacterial peritonitis in patients with cirrhosis. J Gastroenterol Hepatol.

[27] Mandorfer M, Bota S, Schwabl P, Bucsics T, Pfisterer N, Summereder C, Hagmann M, Blacky A, Ferlitsch A, Sieghart W, Trauner M, Peck-Radosavljevic M, Reiberger T (2014). Proton pump inhibitor intake neither predisposes to spontaneous bacterial peritonitis or other infections nor increases mortality in patients with cirrhosis and ascites. PLoS One.

[28] Lewis DS, Lee TH, Konanur M, Ziegler C, Hall MD, Pabon-Ramos WM, Suhocki PV, Smith TP, Kim CY, Choi SS, Ronald J (2019). Proton pump inhibitor use is associated with an increased frequency of new or worsening hepatic encephalopathy after transjugular intrahepatic portosystemic shunt creation. J Vasc Interv Radiol.

[29] Tsai CF, Chen MH, Wang YP, Chu CJ, Huang YH, Lin HC, Hou MC, Lee FY, Su TP, Lu CL (2017). Proton pump inhibitors increase risk for hepatic encephalopathy in patients with cirrhosis in a population study. Gastroenterology.

[30] Bajaj JS, Reddy KR, Tandon P, Wong F, Kamath PS, Garcia-Tsao G, Maliakkal B, Biggins SW, Thuluvath PJ, Fallon MB, Subramanian RM, Vargas H, Thacker LR, O'Leary JG, North American Consortium for the Study of End-Stage Liver Disease (2016). The 3-month readmission rate remains unacceptably high in a large North American cohort of patients with cirrhosis. Hepatology.

[31] Bajaj JS, Liu EJ, Kheradman R, Fagan A, Heuman DM, White M, Gavis EA, Hylemon P, Sikaroodi M, Gillevet PM (2018). Fungal dysbiosis in cirrhosis. Gut.

[32] Kwon JH, Koh SJ, Kim W, Jung YJ, Kim JW, Kim BG, Lee KL, Im JP, Kim YJ, Kim JS, Yoon JH, Lee HS, Jung HC (2014). Mortality associated with proton pump inhibitors in cirrhotic patients with spontaneous bacterial peritonitis. J Gastroenterol Hepatol.

[33] Hung TH, Lee HF, Tseng CW, Tsai CC, Tsai CC (2018). Effect of proton pump inhibitors in hospitalization on mortality of patients with hepatic encephalopathy and cirrhosis but no active gastrointestinal bleeding. Clin Res Hepatol Gastroenterol.

[34] Xie Y, Bowe B, Li T, Xian H, Yan Y, al-Aly Z (2017). Risk of death among users of proton pump inhibitors: a longitudinal observational cohort study of United States veterans. BMJ open.

[35] Xie Y, Bowe B, Yan Y, Xian H, Li T, al-Aly Z (2019). Estimates of all cause mortality and cause specific mortality associated with proton pump inhibitors among US veterans: cohort study. BMJ.

[36] Tvingsholm SA, Dehlendorff C, Osterlind K (2018). Proton pump inhibitor use and cancer mortality. Int J Cancer.

[37] Hsu YC, Lin JT, Chen TT, Wu MS, Wu CY (2012). Long-term risk of recurrent peptic ulcer bleeding in patients with liver cirrhosis: a 10-year nationwide cohort study. Hepatology.

[38] Luo JC, Leu HB, Hou MC, Huang CC, Lin HC, Lee FY, Chang FY, Chan WL, Lin SJ, Chen JW (2012). Cirrhotic patients at increased risk of peptic ulcer bleeding: a nationwide population-based cohort study. Aliment Pharmacol Ther.

[39] Kirk AP, Dooley JS, Hunt RH (1980). Peptic ulceration in patients with chronic liver disease. Dig Dis Sci.

[40] Lenti MV, Pasina L, Cococcia S, Cortesi L, Miceli E, Caccia Dominioni C, Pisati M, Mengoli C, Perticone F, Nobili A, di Sabatino A, Corazza GR (2019). Mortality rate and risk factors for gastrointestinal bleeding in elderly patients. Eur J Intern Med.

[41] Holland-Bill L, Christiansen CF, Gammelager H, Mortensen RN, Pedersen L, Sørensen HT (2015). Chronic live disease and 90-day mortality in 21359 patients following peptic ulcer bleeding – a nationwide cohort study. Aliment Pharmacol Ther.

[42] Leontiadis GI, Molloy-Bland M, Moayyedi P, Howden CW (2013). Effect of comorbidity on mortality in patients with peptic ulcer bleeding: systematic review and meta-analysis. Am J Gastroenterol.

[43] Marmo R, Koch M, Cipolletta L, Capurso L, Pera A, Bianco MA, Rocca R, Dezi A, Fasoli R, Brunati S, Lorenzini I, Germani U, di Matteo G, Giorgio P, Imperiali G, Minoli G, Barberani F, Boschetto S, Martorano M, Gatto G, Amuso M, Pastorelli A, Torre ES, Triossi O, Buzzi A, Cestari R, Della Casa D, Proietti M, Tanzilli A, Aragona G, Giangregorio F, Allegretta L, Tronci S, Michetti P, Romagnoli P, Nucci A, Rogai F, Piubello W, Tebaldi M, Bonfante F, Casadei A, Cortini C, Chiozzini G, Girardi L, Leoci C, Bagnalasta G, Segato S, Chianese G, Salvagnini M, Rotondano G (2008). Predictive factors of mortality from nonvariceal upper gastrointestinal hemorrhage: a multicenter study. Am J Gastroenterol.

[44] Ardevol A, Ibanez-Sanz G, Profitos J (2018). Survival of patients with cirrhosis and acute peptic ulcer bleeding compared with variceal bleeding using current first-line therapies. Hepatology.

[45] Shaheen NJ, Stuart E, Schmitz SM, Mitchell KL, Fried MW, Zacks S, Russo MW, Galanko J, Shrestha R (2005). Pantoprazole reduces the size of postbanding ulcers after variceal band ligation: a randomized, controlled trial. Hepatology.

[46] Lo EAG, Wilby KJ, Ensom MHH (2015). Use of proton pump inhibitors in the management of gastroesophageal varices: a systematic review. Ann Pharmacother.

[47] Garcia-Saenz-de-Sicilia M, Sanchez-Avila F, Chavez-Tapia NC, Lopez-Arce G, Garcia-Osogobio S, Ruiz-Cordero R, Tellez-Avila FI (2010). PPIs are not associated with a lower incidence of portal hypertension-related bleeding in cirrhosis. World J Gastroenterol.

[48] Weersink RA, Bouma M, Burger DM, Drenth JPH, Harkes-Idzinga SF, Hunfeld NGM, Metselaar HJ, Monster-Simons MH, van Putten SAW, Taxis K, Borgsteede SD (2018). Safe use of proton pump inhibitors in patients with cirrhosis. B J Clin Pharmacol.

[49] Haastrup PF, Thompson W, Sondergaard J (2018). Side effects of long-term proton pump inhibitor use: a review. Basic Clin Pharmacol Toxicol.

[50] Scaglione SJ, Metcalfe L, Kliethermes S, Vasilyev I, Tsang R, Caines A, Mumtaz S, Goyal V, Khalid A, Shoham D, Markossian T, Luke A, Underwood H, Cotler SJ (2017). Early hospital readmissions and mortality in patients with decompensated cirrhosis enrolled in a large national health insurance administrative database. J Clin Gastroenterol.

[51] Moon AM, Hayashi PH, Barritt AS (2019). Letter to the editor: Proton pump inhibitors in cirrhosis: a marker of morbid conditions or a cause of mortality. Hepatology.

[52] Chen YJ, Tsai CF, Wang YP, Lu CL (2019). Letter to the editor: proton pump inhibitors are associated with minimal and overt hepatic encephalopathy and increased mortality in patients with cirrhosis. Hepatology.

[53] Waldum HL, Qvigstad G, Fossmark R, Kleveland PM, Sandvik AK (2010). Rebound acid hypersecretion from a physiological, pathophysiological and clinical viewpoint. Scand J Gastroenterol.

[54] Lodrup AB, Reimer C, Bytzer P (2013). Systematic review: symptoms of rebound acid hypersecretion following pump inhibitor treatment. Scand J Gastroenterol.

[55] Haastrup P, Paulsen MS, Begtrup LM, Hansen JM, Jarbol DE (2014). Stategies for discontinuation of proton pump inhibitors: a systematic review. Fam Pract.

[56] Volk ML, Tocco RS, Bazick J, Rakoski MO, Lok AS (2012). Hospital readmissions among patients with decompensated cirrhosis. Am J Gastroenterol.

[57] Moreau R, Jalan R, Gines P, Pavesi M, Angeli P, Cordoba J, Durand F, Gustot T, Saliba F, Domenicali M, Gerbes A, Wendon J, Alessandria C, Laleman W, Zeuzem S, Trebicka J, Bernardi M, Arroyo V, CANONIC Study Investigators of the EASL–CLIF Consortium (2013). Acute-on-chronic liver failure is a distinct syndrome that develops in patients with acute decompensation of cirrhosis. Gastroenterology.

[58] Fischbach W, Malfertheiner P, Lynen Jansen P, et al. S2k-guideline helicobacter pylori and gastroduodenal ulcer disease. Z Gastroenterol. 2017;55:167–206. 10.1055/s-0042-119653. 10.1055/s-0042-119653 27919112

[59] Koop H, Fuchs KH, Labenz J, Lynen Jansen P, Messmann H, Miehlke S, Schepp W, Wenzl TG, Mitarbeiter der Leitliniengruppe (2014). S2k guideline: gastroenterological reflux disease guided by the German society of gastroenterology: AWMF register no. 021-013. Z Gastroenterol.

[60] Götz M, Anders M, Biecker E, Bojarski C, Braun G, Brechmann T, Dechêne A, Dollinger M, Gawaz M, Kiesslich R, Schilling D, Tacke F, Zipprich A, Trebicka J, Deutsche Gesellschaft für Gastroenterologie, Verdauungs- und Stoffwechselkrankheiten (DGVS) (federführend), Deutschen Morbus Crohn und Colitis ulcerosa Vereinigung (DCCV), Deutsche Röntgengesellschaft (DRG), Deutsche Gesellschaft für interventionelle Radiologie (DeGiR), Deutsche Gesellschaft für Allgemein- und Viszeralchirurgie (DGAV) und Chirurgische Arbeitsgemeinschaft für Endoskopie und Sonographie (CAES) der DGAV, Deutsche Gesellschaft für Internistische Intensivmedizin (DGIIN), Deutsche Gesellschaft für Innere Medizin (DGIM), Deutsche Gesellschaft für Kardiologie (DGK), Akademie für Ethik in der Medizin (AEM), Gesellschaft für Thrombose- und Hämostaseforschung (GTH), Collaborators (2017). S2k guideline gastrointestinal bleeding – guideline of the German society of Gastroenterology DGVS. Z Gastroenterol.

